# Supplementary material for: Induced neural phase precession through exogenous electric fields
Source: Nat Commun. 2024 Feb 24;15:1687. doi: 10.1038/s41467-024-45898-5 (PMC10894208; doi:10.1038/s41467-024-45898-5)
Supplement: Supplementary file 2 — Reporting Summary [file 41467_2024_45898_MOESM2_ESM.pdf]

nature portfolio

Corresponding author(s): Miles Wischnewski

Last updated by author(s): Jan 8, 2024

Reporting Summary

Nature Portfolio wishes to improve the reproducibility of the work that we publish. This form provides structure for consistency and transparency in reporting. For further information on Nature Portfolio policies, see our Editorial Policies and the Editorial Policy Checklist.

Statistics

For all statistical analyses, confirm that the following items are present in the figure legend, table legend, main text, or Methods section.

n/a	Confirmed	
☐	☒	The exact sample size (*n*) for each experimental group/condition, given as a discrete number and unit of measurement
☐	☒	A statement on whether measurements were taken from distinct samples or whether the same sample was measured repeatedly
☐	☒	The statistical test(s) used AND whether they are one- or two-sided<br>*Only common tests should be described solely by name; describe more complex techniques in the Methods section.*
☐	☒	A description of all covariates tested
☐	☒	A description of any assumptions or corrections, such as tests of normality and adjustment for multiple comparisons
☐	☒	A full description of the statistical parameters including central tendency (e.g. means) or other basic estimates (e.g. regression coefficient) AND variation (e.g. standard deviation) or associated estimates of uncertainty (e.g. confidence intervals)
☐	☒	For null hypothesis testing, the test statistic (e.g. *F*, *t*, *r*) with confidence intervals, effect sizes, degrees of freedom and *P* value noted<br>*Give P values as exact values whenever suitable.*
☒	☐	For Bayesian analysis, information on the choice of priors and Markov chain Monte Carlo settings
☒	☐	For hierarchical and complex designs, identification of the appropriate level for tests and full reporting of outcomes
☐	☒	Estimates of effect sizes (e.g. Cohen's *d*, Pearson's *r*), indicating how they were calculated

Our web collection on statistics for biologists contains articles on many of the points above.

Software and code

Policy information about availability of computer code

Data collection	Collection of data was done through system-related programs. Specifically, BIOPAC systems, Inc., Goleta, CA, USA was used for EMG recording. StarStim 8 system (Neuroelectrics®, Cambridge, MA) was used for tACS using circular Ag/AgCl electrodes with 1 cm radius (Pistim; 3.14 cm2). Magventure, MagPro X100 (Farum, Denmark) was used for TMS using a Cool-B65 figure-of-eight coil. Neuron recordings in the non-human primate were made using a 128-channel headstage (SpikeGadgets, San Francisco, CA, USA)
Data analysis	Matlab version 2020b/2021b was used for data analysis. Matlab 2020b/2021b was used for statistical analysis using the statistics and machine learning toolbox. For NHP spiking data analysis open-source and freely available toolboxes Wave_clus and Fieldtrip were used. For computational modeling was done in Python using open-source and freely available toolboxes NetPyNE and NEURON.

For manuscripts utilizing custom algorithms or software that are central to the research but not yet described in published literature, software must be made available to editors and reviewers. We strongly encourage code deposition in a community repository (e.g. GitHub). See the Nature Portfolio guidelines for submitting code & software for further information.

Data

Policy information about availability of data

All manuscripts must include a data availability statement. This statement should provide the following information, where applicable:

- Accession codes, unique identifiers, or web links for publicly available datasets
- A description of any restrictions on data availability
- For clinical datasets or third party data, please ensure that the statement adheres to our policy

Experimental human data is available upon a request to the principal investigator according to the IRB protocol approved by the University of Minnesota. Experimental animal data is available upon a request to the principal investigator according to the IACUC protocol approved by the University of Minnesota.

Research involving human participants, their data, or biological material

Policy information about studies with human participants or human data. See also policy information about sex, gender (identity/presentation), and sexual orientation and race, ethnicity and racism.

Reporting on sex and gender	We included 20 healthy volunteers (9 female/11 male). Sex was based on self-report.
Reporting on race, ethnicity, or other socially relevant groupings	No information on race, ethnicity or socially relevant grouping was collected.
Population characteristics	All participants were between 18 and 45 years of age, right-handed, and without a history of I) epilepsy of seizures, II) neurological or psychiatric disorders, III) head injuries, or IV) metal or electric implants in the head, neck, or chest area.
Recruitment	Recruitment was through advertisements at the campus on the University of Minnesota. The sample therefore randomly drew from a general university population (e.g., students and researchers). All volunteers who reached out and were conform the inclusion criteria were included. Potential biases therefore include socio-economic status and educational background.
Ethics oversight	All volunteers gave written informed consent prior to participation. The study was approved by the institutional review board of the University of Minnesota.

Note that full information on the approval of the study protocol must also be provided in the manuscript.

Field-specific reporting

Please select the one below that is the best fit for your research. If you are not sure, read the appropriate sections before making your selection.

☒ Life sciences ☐ Behavioural & social sciences ☐ Ecological, evolutionary & environmental sciences

For a reference copy of the document with all sections, see nature.com/documents/nr-reporting-summary-flat.pdf

Life sciences study design

All studies must disclose on these points even when the disclosure is negative.

Sample size	Human data: N = 20, based on a previous study (Wischnewski et al., 2022). Based on this previous study an effect size of approximately d = 0.6 was anticipated. A power analysis in G*Power 3 using an alpha value of 0.05 and power (1-beta) of 0.80 showed that a sample of n=17 is required. To cover potential drop out or missing data a sample size of n=20 was collected Animal data: N = 81 neurons were identified. This sample size is consistent with similar studies (Johnson, Alekseichuk et al., 2020, Krause et al., 2019).
Data exclusions	No data was excluded
Replication	In human and animal experiment each experimental session consisted of four identical blocks in which phase-dependency was investigated using tACS and TMS. Results are consistent across blocks (see supplementary data). No replication with an independent sample was planned as part of this protocol.
Randomization	Volunteers participated in two sessions, where alpha and beta stimulation was applied. The order of sessions was randomized. Phase specific stimulation during each experiment was also randomized. There were no relevant covariates. Stimulation order for the animal experiment was randomized and counter-balanced.
Blinding	In the human experiment both investigator and participants were blinded to the order of phase-specific stimulation, both during collection and analysis. In the animal experiment the Investigator blinding was not possible as the investigator needed to operate the stimulation device.

Reporting for specific materials, systems and methods

We require information from authors about some types of materials, experimental systems and methods used in many studies. Here, indicate whether each material, system or method listed is relevant to your study. If you are not sure if a list item applies to your research, read the appropriate section before selecting a response.

Materials & experimental systems

n/a	Involved in the study
☒	☐ Antibodies
☒	☐ Eukaryotic cell lines
☒	☐ Palaeontology and archaeology
☐	☒ Animals and other organisms
☒	☐ Clinical data
☒	☐ Dual use research of concern
☒	☐ Plants

Methods

n/a	Involved in the study
☒	☐ ChIP-seq
☒	☐ Flow cytometry
☒	☐ MRI-based neuroimaging

Animals and other research organisms

Policy information about studies involving animals; ARRIVE guidelines recommended for reporting animal research, and Sex and Gender in Research

Laboratory animals	Data used in this study were collected from one rhesus macaque (Macaca mulatta, 13.5kg, 9 years old, male).
Wild animals	No wild animals were used.
Reporting on sex	Male
Field-collected samples	No field-collected samples were used.
Ethics oversight	All animal procedures described here were approved by the Institutional Animal Care and Use Committee of the University of Minnesota (IACUC) and were conducted in accordance with the Public Health Service policy on Humane Care and Use of Laboratory Animals.

Note that full information on the approval of the study protocol must also be provided in the manuscript.
